# A review of descending pain modulation in humans

**DOI:** 10.3389/fpain.2026.1743583

**Published:** 2026-06-04

**Authors:** Christopher D’Souza, Eden Brander-Whittingham, Shima Hassanpour, Brieana Keast, Jessica Merletti, Matthew Kong, Hannan Algitami, Patrick Stroman

**Affiliations:** 1 Centre for Neuroscience Studies, Queen’s University, Kingston, ON, Canada; 2 Centre for Neuroscience Studies, Dept. of Biomedical and Molecular Sciences, Queen’s University, Kingston, ON, Canada

**Keywords:** chronic pain, descending pain inhibition, functional connectivity (FC), nociception, pain, pain inhibition

## Abstract

Descending pain regulation has been widely studied in animals and recent studies in humans have offered new insights into this complex mechanism. This review paper aims to investigate this process in humans to better understand how pain is modulated in the body. This review included primary articles from PubMed related to pain modulation in the human brain or spinal cord. Book chapters, sources using animal models, and review papers were excluded from the paper. Findings consistently noted the periaqueductal grey and rostral medulla as central hubs of descending pain modulation, while cortical regions including the prefrontal cortex, anterior cingulate cortex, insula, and amygdala exhibited variable and context-dependent contributions. Cognitive and emotional processes, including distraction, imagery, music, catastrophizing, and anxiety, were found to modulate descending pain pathways via altered connectivity between cortical and brainstem regions. Studies also identified therapeutic applications, with transcranial direct current stimulation and spinal cord stimulation demonstrating effects on descending modulatory circuits. These studies identify exciting new progress in our understanding of pain modulation whilst also highlighting a need for further study in human populations to clarify certain inferences.

## 1 Introduction

Descending pain modulation is a complex mechanism, involving a balance in activity between nociceptive and anti-nociceptive pathways. These circuits enable the modulation of pain in both inhibitory and facilitatory directions, influencing pain perception in healthy and clinical populations (1). While animal studies have laid the foundational knowledge in this field, human neuroimaging, neuropsychological, and clinical studies have expanded our understanding of the mechanisms of descending modulation in humans. This review examines primary human studies to explore how descending pain pathways function normally and under different conditions, to provide a clearer picture of the functional architecture and clinical relevance of descending pain modulation in humans.

## 2 Methods

The articles used in this study were collected from PubMed using the following search terms: (“descending pain”) AND (modulation OR regulation) AND (Human) AND (brain OR brainstem OR cord) NOT (review). Papers that were not primary sources, did not involve human subjects, and duplicates were excluded from the review, as well as any works that did not comment on the mechanisms of descending pain. During the review process, all references were organized in Covidence and were reviewed by two authors each to reduce bias. Papers underwent an initial title and abstract screening before proceeding to full text screening in order to judge eligibility criteria. All papers were screened by two authors to reduce bias when excluding studies. Papers that fulfilled these criteria were assembled and divided into the four sections in this review, with studies not being exclusive to one section.

## 3 Results

### 3.1 Study selection

A total of 156 studies were collected from PubMed and screened for this review. 6 duplicate records were identified by Covidence and were excluded from the review process, and the remaining 150 studies proceeded to title and abstract screening. 27 studies were excluded through title and abstract screenings as they included animal models and therefore did not meet the inclusion criteria for the review. 123 studies underwent full text screening and a total of 37 were excluded. Of these, 5 studies were excluded due to inappropriate study design such as textbook chapters or review articles. The other 32 papers were excluded due to not focusing sufficiently on descending pain modulation.

**Figure 1:**
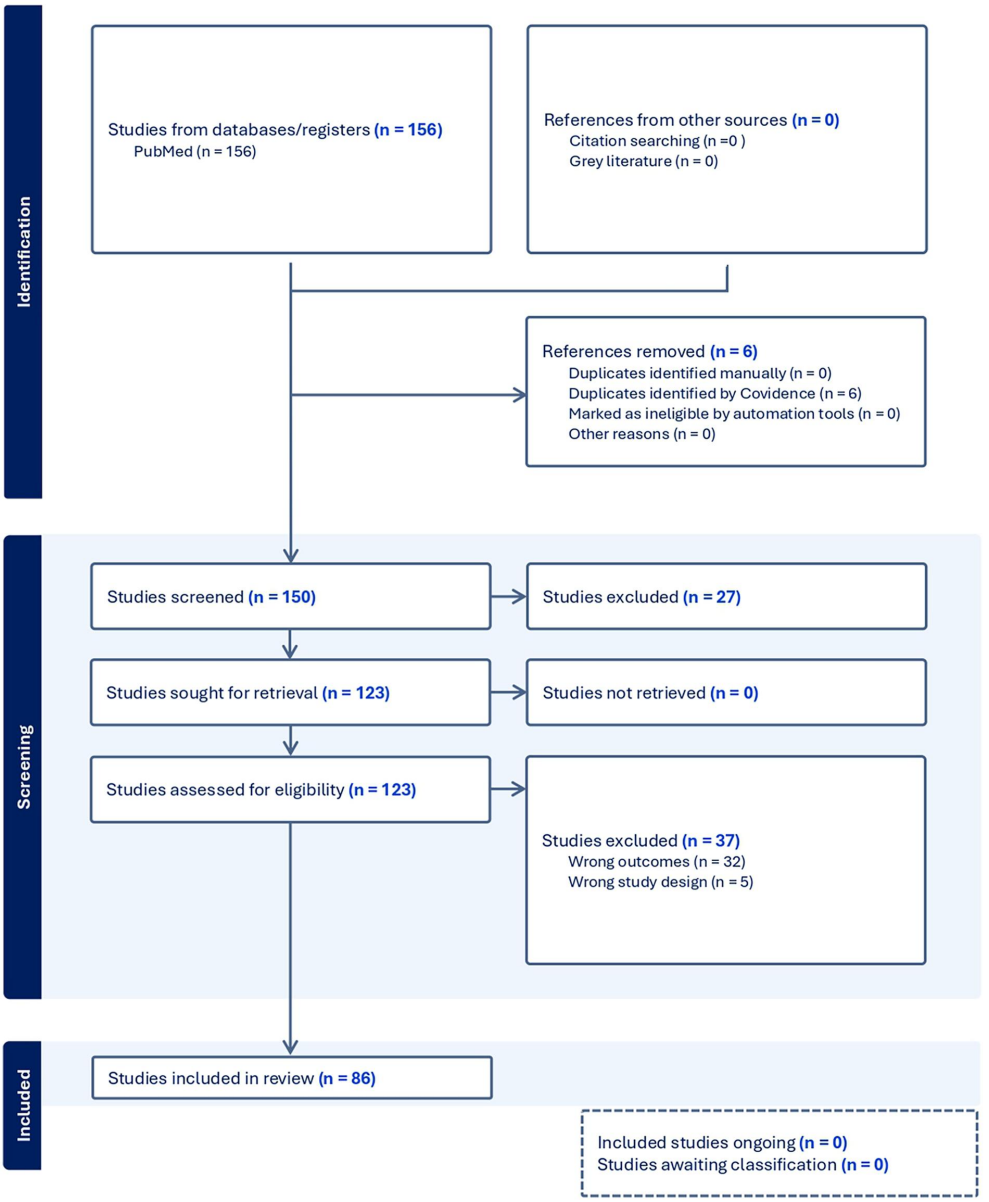
PRISMA flow diagram, detailing how many studies were included and excluded from the review process and for what reason.

After the screening process, 86 studies met the inclusion criteria and were included in the final review of the analysis. No ongoing studies or ones awaiting classification were included in the review. The study selection process is summarized in the PRISMA flow diagram (Figure 1).

All studies underwent a full text review and were organized into four sections, according to the predominant themes across all papers. These include functional connectivity in descending pain networks, psychological factors influencing pain modulation, neurotransmitters involved in descending pain regulation, and clinical factors associated with alterations in descending pain modulation.

### 3.2 Functional connectivity in descending pain structures

**Figure 2:**
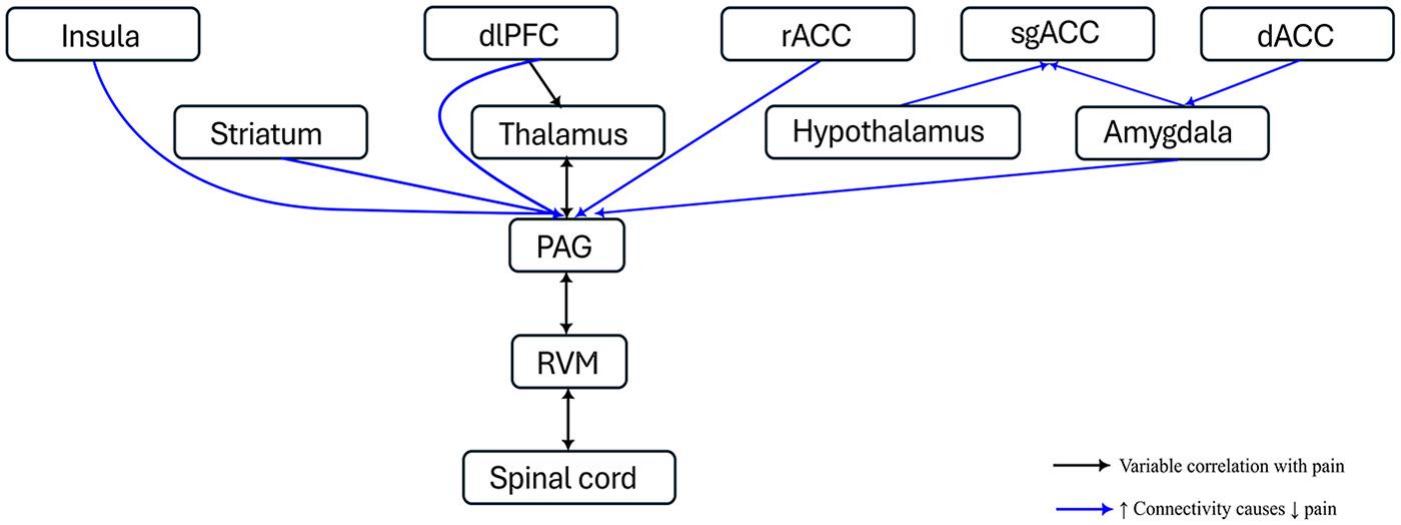
A summary of functional connectivity in important brain regions whose connections reduce pain.

**Figure 3:**
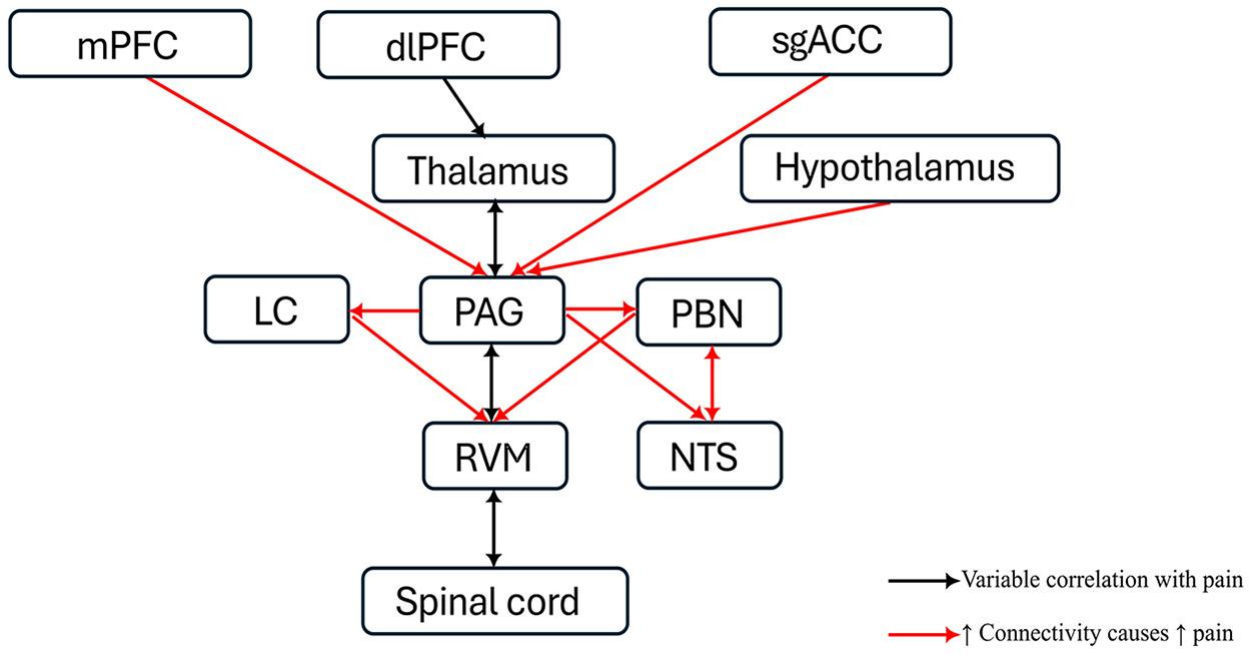
A summary of functional connectivity in important brain regions whose connections increase pain.

Building on foundational knowledge of the neuroanatomy and physiology behind nociceptive signaling and pain processing, recent studies have provided valuable insights into the mechanisms of descending pain modulation. The reviewed works expand our existing understanding by explaining how these systems operate in humans and how they may be altered in the context of disease. An overview of the primary functional connections described in this section is provided in Figures 2 and 3, with Figure 2 summarizing connections associated with pain inhibition and Figure 3 summarizing connections associated with pain facilitation.

#### 3.2.1 The periaqueductal grey and rostral medulla

Located in the midbrain, the periaqueductal grey (PAG) plays a critical role in controlling descending pain modulation by exerting top-down control over spinal nociceptive processing (2–4). Functional connectivity (FC) studies support the idea that the PAG downregulates pain, demonstrating an inverse relationship between PAG activation and pain ratings (5, 6). Importantly, FC reflects coordinated activity between brain regions rather than direct anatomical connection and observed interactions are likely mediated through polysynaptic pathways. Although descending outputs are mediated through serotonergic and noradrenergic systems, opioidergic signaling within the PAG is thought to modulate this network by inhibiting ascending pathways, triggering analgesia (2, 7). Tinnermann et al. found that opioid agonist treatment with remifentanil was associated with increases in PAG to spinal cord functional connectivity, supporting the idea that the PAG exerts control via opioid signaling (2). Furthermore, when opioid receptors are activated, the blood oxygen level dependent (BOLD) response in the dorsal horn is dampened, suggesting that the release of opioids inhibits ascending pain transmission, triggering analgesia (2, 7).

The PAG receives inputs from somatotopically organized ascending pain fibers in the dorsal horn of the spinal cord (2, 7). Mehnert et al., provide evidence of this in an fMRI study where they stimulated branches of the trigeminal and occipital nerves. They found that the C2 and C3 dermatomes, located on the posterior scalp and neck, were represented by clusters in the most cranial parts of the PAG. In contrast, regions innervated by trigeminal nerve branches (V1-3) located in the face, are represented in descending clusters organized from cranial to caudal (7). This information is also relayed to the primary somatosensory cortex as demonstrated by functional connectivity studies, leading the conscious feeling of pain (8).

Pain modulation by the PAG is underpinned by its bidirectional connectivity with the rostral ventromedial medulla (RVM) (2, 5, 6, 9–11). While the term RVM originates from studies on animal models, recent findings suggest the term's description might not be as accurate in humans (12). Located in the upper (rostral) medulla, the RVM receives input from and projects to the PAG as well as the dorsal horn of the spinal cord, acting as the relay from the PAG to the spinal cord (2, 9, 10). Pain studies have observed an increase in RVM activity in correlation with higher analgesia, supporting the belief that the region is responsible for dampening pain (9). Furthermore, Eippert et al., found that participants treated with naloxone had reductions in placebo analgesia and decreased BOLD responses in the PAG and RVM, confirming that it acts via opioid signaling (13). Regions of the RVM include the serotonergic nuclei in the nucleus raphe magnus (NRM) and the medullary reticular nuclei in the nucleus gigantocellularis (NGc); both of which have been shown to have important roles in descending pain regulation (5, 6). The NRM receives inputs from the locus coeruleus, PAG and parabrachial nucleus and projects to the dorsal horn and continuously downregulates pain (6). Stroman et al.` reported BOLD signal variations in the NRM before participants were presented with a noxious stimulus, suggesting that the NGc contributes to pain regulation in response to nociceptive input, rather than anticipatory modulation (5). The NGc also projects to the dorsal horn of the spinal cord where its function is believed to downregulate pain sensitivity (5, 6). Functional connectivity studies have observed BOLD signal changes in the NGc scaling with participant's pain ratings, suggesting a reactive role in pain regulation, however, further studies are needed to confirm this (5, 6). Together, the PAG and RVM form a critical pathway for descending pain modulation, integrating top-down signals to regulate nociceptive processing and maintain pain homeostasis.

#### 3.2.2 Thalamus

The thalamus plays a unique role in the pain modulation circuit. It receives ascending inputs from the dorsal horn of the spinal cord and projects broadly to cortical regions, including the primary and secondary somatosensory cortices and the primary motor cortex (14–18). The thalamus is believed to serve as a conduit through which different pain modulation pathways converge, while the structure itself is not a primary effector of descending pain regulation (15, 19).

Functional connectivity studies support this belief. Zhou et al. reported FC between the thalamus and vlPAG and studies in migraine populations also have observed the same connection correlating positively with pain intensity (20, 21). However, work by Li et al. in a separate study examining migraines severity reported that FC between these two regions was negatively correlated with pain intensity, indicating the relationship between these regions may be context dependent. The thalamus receives information from cortical regions such as the dlPFC and the ACC which exert variable influences on descending pain modulation and may contribute to the contradictory findings between Li and Zhou's findings (19, 20). FC between the thalamus and PAG reflects coordinated activity rather than a direct anatomical projection and thalamic influence over PAG-mediated pain modulation is likely occurring through indirect, polysynaptic pathways. Therefore, the reviewed works suggest that the thalamus functions as an intermediary hub, linking cortical and brainstem circuits that contribute to descending pain modulation (20).

#### 3.2.3 Hypothalamus

The reviewed articles suggest the hypothalamus downregulates pain modulation through the hypothalamic-pituitary-adrenal (HPA) axis, its connectivity with periaqueductal gray (PAG) and other brain regions. A study by Mainero et al., found that the hypothalamus-PAG FC increases with migraine severity and frequency suggesting that the hypothalamus influences the progression of chronic migraines, increasing pain (21). Furthermore, Staud et al. found PAG-hypothalamus FC increased significantly fibromyalgia patients compared to healthy controls, suggesting dysregulated descending pain inhibition, and an increased stress response to pain (6). Moreover, in cluster headaches (CH), the hypothalamus exhibits a more distinct role. FDG-PET imaging revelated hypothalamic activity does not remain elevated during CH attacks, indicating that its primary function lies in initiation rather than ongoing pain processing (22). Together, these findings suggest that the hypothalamus heightens pain sensitivity by influencing descending control networks and stress systems, particularly in chronic pain states.

#### 3.2.4 Prefrontal cortex

The prefrontal cortex (PFC) plays a complex and multitasking role in helping regulate descending pain modulation. It is divided into the lateral PFC, consisting of the dorsolateral PFC (dlPFC) and ventrolateral PFC (vlPFC); and the medial PFC which includes the ventromedial PFC (vmPFC) (23). These regions make up many different pathways involved in descending pain modulation.

The dlPFC modulates the cognitive and perceptional aspects of descending pain modulation (3, 20, 24, 25). This theory is supported by Zhou, et al. and Jin et al. who found weakening attention to noxious stimuli, caused by acupuncture therapy would decrease rDLPFC – vlPAG connectivity and increase analgesia (20, 26). Stein et al. also note a correlation between the integrity of white matter tracts connecting the dlPFC and PAG and pain sensitivity, further supporting this belief (27). The reviewed works suggest that the dlPFC modulates pain by inhibiting ascending pain pathways which travel from the midbrain to the thalamus to the cingulate cortex (22). FC studies have supported these findings, noting that the dlPFC – dACC connection is positively associated with PAG activity and placebo analgesia. Based on these findings, it is hypothesized that the dlPFC initiates descending pain regulation through projections to the dACC which in turn activates the PAG to inhibit pain perception (3, 27). Many of the reviewed articles also identified that the dlPFC has direct connections to the PAG, with increased dlPFC – PAG FC correlating with lower analgesic response (3, 20, 27–29). Work by Wang et al. also identified connections between the dlPFC and the posterior thalamus which is active during prolonged bouts of pain in migraine patients. A positive correlation between dlPFC – thalamus and pain intensity suggested the connection heightens individual's awareness of pain, contributing to the overall narrative of the dlPFC (30).

The medial prefrontal cortex (mPFC) plays a similar role in descending pain modulation as the dlPFC, regulating the cortical aspect of pain, with activity in the region correlating with higher pain ratings (28). The mPFC is seen to be primarily connected to the PAG, and greater FC between these regions is typically correlated with higher pain ratings in chronic pain studies (4, 9, 31). Furthermore, work by Li et al. reported FC between the posterior cingulate gyrus and the mPFC which is also positively related to pain intensity (19). However, the assumption that the mPFC is tied to higher pain intensity might be an overgeneralization. A study by Lin et al. on patients with chronic pain found activity in the right mPFC correlated to higher analgesia, suggesting the function of the mPFC in descending pain modulation may be more flexible than previously believed (32). The ventromedial PFC (vmPFC) is a subdivision of the mPFC which projects to the PAG and the dlPFC (9, 24, 33). The primary theory regarding the vmPFC and its role in descending pain is it projects to the PAG to inhibit pain. However, contradictory findings show the vmPFC disrupts the connection between the PAG – lateral PFC, facilitating pain instead (33). Due to a lack of supporting evidence, the exact role of this connection is difficult to clearly determine and will require further investigation.

#### 3.2.5 Anterior cingulate cortex

The cingulate cortex is an important input region which influences descending pain regulation, relaying information from higher cortical areas (14, 34). The subgenual anterior cingulate cortex (sgACC) - PAG FC is prominent during tonic pain tests, with increased connectivity correlating to higher pain ratings (14, 35). Furthermore, Cheng et al. also identified a negative correlation between sgACC – RVM functional connectivity in a study examining the temporal summation of pain effect (36). Nickel et al. theorize the PAG can exert facilitatory or inhibitory control over pain modulation and input from the sgACC during prolonged periods of pain leads to more inhibition and greater pain ratings (35). The sgACC receives inputs from the hypothalamus and amygdala via separate pathways and projects to the PAG (34, 37). The hypothalamic pathway is thought to be related to the sympathetic response due to its involvement in the sympathomedullary pathway while the amygdala is believed to be involved in the emotional regulation of pain (14, 34, 37). Furthermore, sex difference studies found women demonstrate stronger FC between the sgACC and PAG while men exhibit stronger structural connectivity between the sgACC and hypothalamus which could imply biological men and women favor different descending pain pathways (11, 34, 37).

Expectation and attention to pain stimuli also affects the level of PAG activation which is believed to be regulated by the rostral anterior cingulate cortex (rACC). The rACC – PAG connection is positively associated with attenuation of pain, implying the pathway promotes descending pain regulation by exciting the PAG during prolonged exposure to nociceptive stimuli (4, 33, 38–41). A study examining expectation of pain found that negative expectation (anticipating more pain), led to weaker rACC – PAG FC and subsequently higher pain ratings (38). Furthermore, studies on chronic pain conditions observed reductions in FC between these two regions in patients with acute pain conditions such as migraine, providing supporting evidence that the pathway plays a role in pain regulation (8, 42). Many of the reviewed works also suggest acupuncture may be an effective treatment due to its ability to strengthen the rACC-PAG connection, helping to restore pain regulation (4, 33, 43).

The dorsal anterior cingulate cortex (dACC) is known to be part of the medial pain processing pathway, a network involved in the cognitive and affective aspects of pain which specifically integrates pain unpleasantness and suffering (44, 45). This is supported by Vanneste et al. who conducted an EEG study which found increased activation in the dACC, marked by alpha and beta waves, correlated with higher catastrophizing, a maladaptive cognitive pattern characterized by persistent rumination and exaggerated negative expectations about pain (45). Furthermore, laser-evoked potentials in the occipital nerve decreased dACC activation while reducing pain ratings in participants with fibromyalgia, once again supporting the region's involvement in the unpleasantness and discomfort of pain (44).

#### 3.2.6 Amygdala

The amygdala is involved in emotional processing and interacts with other higher order structures of the brain in the descending pain network (10, 46). Work performed by Meulen et al. has demonstrated that the left amygdala receives top-down control from the right ventrolateral prefrontal cortex (vlPFC), and this connection is suggested to be involved in cognitive reappraisal and controlling the perceived threat of a painful stimulus. This study found that the connection decays with age, implying that older adults lose the ability to downregulate the autonomic response to pain (46). The left amygdala also has functional connections to sensorimotor areas in the posterior parietal cortex including S1, S2, and M1 (14). Huynh et al. (2022) provide a potential explanation for this connection, proposing the left amygdala is involved in the affective component of pain and inhibiting nociceptive pathways (47). Meulen et al. also found that the dACC projects to the amygdala as well. Participants who performed memory tasks during nociception showed increased FC between these areas, implying the connection may be related to expectation-based modulation or coping strategies (46). The amygdala also projects to the PAG and ventral tegmental area, and while this connection has been negatively associated with prolonged bouts of chronic pain, it is unclear what the role of this connection plays in descending pain regulation (10, 28, 33, 48).

#### 3.2.7 Insula

The insula is crucial for descending pain modulation, influencing sensory, emotional, and cognitive aspects of pain through its connections with numerous brain regions. Specifically, several studies indicate that its connectivity with the PAG increases descending pain inhibition. Li et al. found that resting state FC between ventrolateral PAG and insula was reduced in individual with migraines, suggesting that a dysfunctional insula-PAG pathway impairs pain inhibition (4). Similarly, older adults exhibited a weaker insula-PAG connectivity, correlating with diminished conditioned pain modulation (CPM), a phenomenon where one painful stimulus reduces the intensity of another painful stimulus. This indicated to researchers that age-related differences in this connection could also lead to reduced pain inhibition (46, 49). Bar et al. provided further insight on the insula's function, noting that the left posterior region increases BOLD signal responses during heightened bouts of pain while the right side does not exhibit this behavior (50). Moreover, the insula's role in expectation-based analgesia was assessed by several studies, finding that positive expectations increased anterior insula-PAG connectivity, while negative expectations disrupted this connection, increasing pain sensitivity (3, 22, 38). Furthermore, activation in the PAG during anticipatory pain trials also seems to trigger increased responses in the posterior insula according to Fairhurst et al., however, the authors state the its role in descending pain remains unclear (41). The administration of gabapentin was found to reduce pain perception in individuals with chronic pain by suppressing posterior insula activity, emphasizing the insula's significance in processing nociceptive inputs and modulating pain inhibition (15). Furthermore, a study observing structural changes from chronic pain due to cluster headaches found increased gray matter volume in the insula. This finding suggests that the insula undergoes neuroplastic changes in response to repeated pain occurrences (51). Collectively, the studies show that dysregulated connectivity between the insular PAG has been observed in migraine, chronic pain, and aging populations, with reduced connectivity being associated with increased pain sensitivity and impaired inhibition.

#### 3.2.8 Parabrachial nucleus and nucleus tractus solitarius

Located in the brainstem, the parabrachial nucleus (PBN) receives inputs from the periphery via the spinal cord dorsal horn (14). According to Stroman et al., the PBN is involved in autonomic regulation of pain, modulating the descending PAG-RVM-spinal cord pathway (5). Functional connectivity studies have identified connections between the PBN and the nucleus tractus solitarius (NTS), RVM (NRM and NGC) and PAG, noting that increased FC between these areas is correlated to greater acute pain ratings (5, 14). Studies on anticipation of pain note that these connections are only present during and after the application of noxious stimuli, leading to the belief that the PBN is not involved in pain expectancy (5, 41). Furthermore, a study conducted on prolonged pain by Meeker et al., reported the PBN FC varies between different regions of the structure. They found that the left PBN showed increased FC with the primary motor cortex, while the right PBN increased connectivity with the somatosensory cortex, near the area that receives afferent input from where the stimulus was applied (14). These findings provide a deeper understanding of the organization of the PBN and highlight its involvement in integrating sensory and motor aspects of prolonged pain.

The NTS, similar to the PBN, is also involved in the autonomic regulation of pain and is functionally connected to the PBN and the PAG (5). Stroman et al. found that the NTS-PAG connectivity increases in periods preceding and during pain and PBN-NTS connectivity increased during stimulation as well. The authors believe this demonstrates that the NTS is involved in continuous regulation of pain by controlling the spinal cord's excitability before noxious stimuli, however, more research is required to confirm this hypothesis (5).

#### 3.2.9 Striatum

Of the articles that were reviewed in this paper, the striatum was sparsely covered, but research indicates its importance in descending pain modulation, primarily through its interactions with the PAG and contribution to pain relief mechanisms. A study conducted by Li et al. identified changes in PAG-striatum connectivity following migraine treatment, involving the nucleus accumbens, ventral striatum, and putamen (4). These structures were correlated with reductions in headache intensity, suggesting that the striatum contributes to analgesia through descending pain modulation. Additionally, a genetic study examining the brain derived neurotrophic factor (BDNF) Val66Met polymorphism found that individuals with the Met/Met variant exhibited increased PAG connectivity with striatal regions and related basal ganglia circuits (28). This is notable because chronic pain has been associated with a shift in pain processing from sensory regions toward limbic circuits, which include the basal ganglia. This mutation reduces the activity of BDNF and therefore these findings imply that genetic predispositions may influence striatal involvement in pain processing, potentially rendering individuals more susceptible to chronic pain (28). Sprenger et al. also reported striatal hypometabolism in chronic headache patients, indicating that disruptions in dopamine-mediated circuits may contribute to pain modulation dysfunction (22).

#### 3.2.10 Locus coeruleus

Located in the brainstem, the locus coeruleus (LC) modulates pain by downregulating nociceptive transmissions from the spinal cord. It receives input from higher cortical regions, including the PFC and ACC, via the PAG and relays these signals to the spinal cord through the NRM (6, 52). This is supported by Staud et al. who report that while experiencing pain, connections from the PAG→LC increased, alongside connections from the LC→NRM and NRM→ spinal cord which are all involved in pain inhibition (6). This highlights a potential cortex→PAG→LC→NRM→ spinal cord pathway, suggesting how cortical processes influence pain perception. The authors also posit that the LC's specific role in pain modulation is through a stress mediated analgesia response as it is known to be part of the reticular activating system which regulates the body's arousal through the release of norepinephrine (6, 53). These findings highlight the pathways the LC is involved in and indicate a potential role in stress-related analgesia.

#### 3.2.11 Future directions

The reviewed works provide excellent details on the roles of major structures involved in the descending pain network. The PAG, PFC, and ACC in particular have been thoroughly explored with studies often supporting each other's findings. However, brainstem regions like the NTS, striatum, and PBN remain relatively unexplored with only a few studies mentioning the structures. Future papers should aim to bridge this gap and improve our knowledge of how these structures and subsequently our understanding of descending pain modulation.

### 3.3 Psychological factors affecting descending pain modulation

It has been shown that descending pain modulation can be influenced by various cognitive and emotional strategies. Mechanisms such as cognitive distraction can affect pain processes through inhibiting pain, while others can further exacerbate it. According to studies on emotional states, spinally mediated nociceptive flexion reflex (RIII) feedback training, such as imagining a safe and happy place, could decrease spinal nociception (52, 54). Dobek et al. performed a study, having participants listen to their favourite music while experiencing noxious stimuli and found pain ratings decreased in participants listening to music (55). Specifically, they note that the dlPFC mediates this process, influencing connectivity between these areas through shifts in attention, emotional regulation, or changes in stimulus valuation (52, 54, 55). Pain inhibition is proposed to occur via direct pathways from the PFC and ACC to the PAG and locus coeruleus, or indirectly through the thalamus. In their model, the LC and PAG, via the RM, reduce nociceptive transmission to the spinal cord, leading to a decreased response in emotional-evaluative cortical regions such as the insula and dlPFC (52). Additionally, Krafft et al. reported that chronic pain patients, particularly those with back pain, have been shown to learn to modulate spinal nociceptive processing through feedback training, which significantly improved their ability to engage descending pain inhibition. The authors observed that the pain-reducing effects of this training were maintained over time, suggesting that learned control over spinal nociception may provide lasting benefits (56). These findings point to the potential for targeted feedback training to enhance pain modulation by modifying nociceptive pathways and improving the brain's ability to control pain perception.

Salience and attention also play crucial roles in pain perception and modulation. According to Yearwood et al., the dACC region has been implicated in the salience of pain, with increased dACC activity drawing more attention to the pain and amplifying its perceived intensity (57). Studies have also shown that attention dysfunction can impair pain modulation. For instance, Yu et al. found that increased functional connectivity between the periaqueductal gray (PAG) and orbital inferior frontal gyrus (IFG) in chronic pain patients may signal dysfunctional pain inhibition, potentially due to attention being overly directed towards pain. The orbital IFG, involved in processing negative emotions and impaired cognitive evaluation of nociceptive information, contributes to this maladaptive focus on pain. Furthermore, the authors report that functional connectivity between the PAG and anterior cingulate cortex correlates negatively with pain intensity, suggesting activation of the descending pain control system (58).

Sex differences have also been observed in the ways that attention influences pain modulation. In a study by Wang et al., the authors found notable differences in the functional connectivity of the subgenual anterior cingulate cortex (sgACC), which plays a key role in emotional and cognitive aspects of pain processing. Their findings showed a marked increase in women for functional connectivity from the sgACC to regions mediating pain habituation, such as the PAG, NRM, medial thalamus, and anterior midcingulate cortex (aMCC), compared to men. In contrast, men displayed stronger functional connectivity from the sgACC to the anterior insula and temporoparietal junction—areas involved in salience detection. These data may suggest that men are capable of more sustained attention to nociceptive stimuli, leading to a reduction in pain habituation (34). Furthermore, women have also shown lower baseline activation of regions involved in descending pain modulation compared to men, which Karshikoff et al. believes demonstrates the pain inhibitory system may be easily disrupted in women (59).

Other cognitive processes, such as catastrophizing, also play a significant role in how pain is experienced and regulated. Pain catastrophizing has been consistently linked to maladaptive pain modulation. Ellingson et al. demonstrated that catastrophizing interferes with the ability to engage cognitive modulation strategies that typically reduce pain in women with fibromyalgia. It is likely that this tendency to catastrophize interferes with the beneficial effects of cognitive distraction on pain modulation, possibly due to its impact on attention-resource allocation, and may contribute to the exacerbation or maintenance of chronic pain (60). These interpretations align with those of Krafft et al., who demonstrated that individuals with chronic back pain who scored higher on measures of catastrophizing exhibited reduced ability to control spinal nociception, suggesting a dysfunction in descending pain pathways (56). The relationship between catastrophizing and pain modulation appears to be complex, as it may not only impair pain inhibition but also enhance pain perception by increasing sensitivity to noxious stimuli. These findings underscore a potential mechanism by which maladaptive cognitive processes can disrupt the normal functioning of pain modulatory systems.

Another cognitive factor that impacts pain modulation is anxiety, which has been shown to impair the effectiveness of descending pain inhibition. According to Vidor et al., individuals with chronic myofascial pain syndrome who exhibited high levels of anxiety showed reduced inhibitory signaling in cortical circuits, suggesting that anxiety disrupts pain inhibitory processes and may enhance pain perception (61). Wiech et al. reported that chronic pain patients often exhibit dysfunctional cortical processing, with reduced activation in regions crucial for pain inhibition, such as the dlPFC. This dysfunction is further compounded by a positive correlation between anxiety and motor cortex excitability, leading to diminished corticospinal modulation of pain (62). Moreover, Li et al. showed similar findings in a study examining neuralgia patients which found a correlation between patients with anxiety and depression and weaker PAG – rACC FC (63). These works suggest anxiety and negative emotions disrupt descending pain pathways, increasing pain. The amygdala, which interacts with opioid mediated pain regulation pathways, may function less effectively in chronic pain patients due to central sensitization, exacerbating pain perception (61). Additionally, anxiety and pain are linked through increased activity in the hippocampal formation (HF), which primes the body to perceive pain more intensely (61). These findings across studies strongly support the idea that anxiety is a key modulator of descending pain regulation, often impairing the capacity of the brain to suppress pain. Nonetheless, the precise neural mechanisms underlying this relationship remain unclear, and future studies are needed to delineate the specific brain networks involved in the interaction between anxiety and pain modulation.

External stimuli, including social and emotional factors, also play an important role in modulating pain, further emphasizing the complexity of pain regulation mechanisms. As seen in the study by Gougeon et al., observing a loved one in pain—or even watching oneself in pain—can trigger descending pain inhibitory processes (64). This suggests that, according to the authors, empathy may activate brain areas involved in pain inhibition, such as the PAG and RM, further illustrating the impact of emotional factors on pain modulation (64). Additionally, the connection between the supramarginal gyrus and the PAG in chronic pain patients may suggest that these individuals experience enhanced empathy. The supramarginal gyrus, which is involved in empathy and social cognition, could play a role in how emotional and social factors influence pain perception (58). Other emotional responses, such as sadness or fear, might interfere with pain inhibition, as indicated by research from Wiech et al., who found that emotional distress can reduce the brain's ability to engage in pain-inhibitory processes and exacerbate the perception of pain (62). Emotions inflicted by other external stimuli have also been shown to influence pain through descending pain-modulatory pathways. In the study by Roy et al., it was found that unpleasant music caused an increase in the nociceptive flexion reflex, involving the removal of an a body part from a noxious stimulus, and pain ratings in participants when compared to being in the presence of pleasant music (65). This further highlights the role that emotional and sensory stimuli can play in modulating pain perception through descending pain pathways.

Descending pain modulation is a complex process influenced by various cognitive, emotional, and external factors. Strategies such as feedback training, attention regulation, and emotional modulation can significantly alter pain perception and inhibition. However, many questions remain regarding the precise neural mechanisms underlying these interactions, particularly in relation to chronic pain conditions. Future research should focus on delineating the specific networks involved across the brain, brainstem, and spinal cord, exploring sex differences, and investigating how maladaptive cognitive patterns like catastrophizing and anxiety can be targeted for therapeutic interventions to improve pain management.

### 3.4 Neurotransmitters and proteins in descending pain modulation

Descending pain modulation is a complex process governed by an interplay between excitatory and inhibitory neurotransmitters, as well as regulatory proteins.

Fanton et al. investigated how translator protein (TSPO) gene variation, specifically the Ala147Thr polymorphism, affects pain regulation and neurotransmitter balance. The TSPO protein is a biomarker of glial cell activation, leading to elevated levels of pro-inflammatory cytokines in the cerebrospinal fluid. The binding of TSPO to its ligand increases pain-related activity in the frontoparietal network, commonly involved in pain expectation. Fanton et al. demonstrate that individuals carrying the high-affinity binding (HAB) variant of the TSPO gene exhibited impaired descending pain inhibition and a reduced capacity for expectancy-induced pain modulation compared to mixed/low-affinity binders (MLABs). These effects led to higher thalamic glutamate concentrations and a positive correlation between glutamate and GABA levels in the rostral anterior cingulate cortex (rACC), suggesting a disruption in excitatory-inhibitory homeostasis. Although these alterations were observed in both fibromyalgia patients and healthy controls, their impact was more pronounced in fibromyalgia due to pre-existing dysfunctions in pain processing. These findings highlight TSPO as a potential contributor to individual differences in pain modulation, possibly through interactions between glial activity, neurotransmitter balance, and expectancy-related pain processing (66).

To explore the role of GABAergic mechanisms in endogenous pain inhibition, Kunz et al. conducted a double-blind, placebo-controlled crossover study examining the effects of lorazepam, a GABAA receptor agonist, on diffuse noxious inhibitory controls (DNIC), usually referred to as CPM in humans, in healthy individuals. The results demonstrated that while lorazepam significantly raised heat pain thresholds, suggesting a general reduction in pain sensitivity, it did not alter the magnitude or pattern of DNIC-mediated pain inhibition. These findings suggest GABAA receptor activation did not significantly alter CPM, although GABAergic signaling remains widespread in pain-related networks (67).

Younis et al. examined pontine glutamatergic metabolism in patients with migraine without aura during the interictal phase using proton magnetic resonance spectroscopy (^1^H-MRS). Despite prior suggestions that glutamate dysregulation contributes to migraine pathophysiology, they found no significant difference in pontine Glx (glutamate plus glutamine) levels between patients and healthy controls. This suggests that excitatory neurotransmission alterations in the pons are unlikely to be a significant factor in migraine without aura. However, other brain regions, such as the thalamus and cortex, may still be involved (68).

Palmer et al. investigated the effects of melatonin on descending pain modulation and neuroplasticity markers in breast cancer patients undergoing their first cycle of chemotherapy. In a randomized, double-blind, placebo-controlled trial, they found that melatonin supplementation significantly improved endogenous pain inhibition, increased heat pain thresholds and tolerances, and reduced serum levels of brain-derived neurotrophic factor (BDNF), tropomyosin receptor kinase B (TrkB), and S100B protein. These changes were independent of melatonin's effects on sleep quality, suggesting a direct modulatory effect on neural plasticity and pain processing (69).

Tarragó et al. explored the relationship between intracortical inhibition and endogenous pain modulation in patients with severe knee osteoarthritis. Using transcranial magnetic stimulation (TMS) and CPM tasks, they found that patients exhibited shorter cortical silent periods (CSPs). CSPs reflect the temporary suppression of voluntary muscle activity after stimulation which indicates reduced GABAergic inhibitory activity, compared to healthy controls. Furthermore, shorter CSP duration was associated with weaker CPM responses, suggesting that impaired inhibitory signaling may contribute to reduced descending pain inhibition. These findings suggest that chronic osteoarthritis pain may be associated with neuroplastic changes that shift cortical networks toward excitability over inhibition, promoting persistent hyperalgesia and disability (70).

Collectively, these studies highlight the importance of regulatory proteins and neurotransmitters in pain control. The TSPO gene and melatonin show promise for future research, however, additional studies are needed to clarify their mechanisms under different conditions. Furthermore, findings regarding the roles of GABA and glutamate in descending pain regulation are mixed. This highlights the need for further research into different disease states and other external factors that may influence these neurotransmitters. It is important to note that many of these signaling molecules, including GABA, glutamate, BDNF, and melatonin-related pathways, are widely distributed throughout the brain and spinal cord (71, 72)**.** Resultingly, experimental findings often reflect larger alterations in pain-processing networks rather than specific effects on specific regions or pathways.

### 3.5 Behavioral and clinical implications

The body of research that was reviewed provides important insights into the mechanisms of descending pain modulation and the potential for targeted interventions in clinical practice. A standout theme is the role of non-invasive brain stimulation to downregulate pathways in the brain facilitating pain and upregulate ones triggering analgesia (53, 73, 74). Transcranial direct current stimulation (tDCS) was the most studied mode of stimulation which uses a low electrical current, administered through electrodes placed on the scalp to modulate brain activity (53).

Authors suggest that tDCS may serve as an effective therapy for chronic pain in conditions such as Parkinson's disease (PD), chronic low back pain (CLBP), and knee osteoarthritis, where deficits in pain modulation add to a diminished quality of life (53, 75, 76). Several studies report that tDCS in the motor or prefrontal cortex enhances the CPM effect over short and long time periods, leading to the belief that descending pain inhibition mechanisms have been restored (53, 77–79). Despite growing evidence supporting the analgesic effects of tDCS, the mechanism by which this intervention functions remains relatively unknown. tDCS studies performed in the primary motor cortex (motor cortex stimulation), hypothesize that the therapy activates cortical regions including the dlPFC and the ACC which are known to exert top-down control over nociceptive processing. This hypothesis aligns with findings that chronic pain conditions including CLBP and fibromyalgia are associated with reduced activity in these regions, however, whether tDCS works by activating pathways involving these regions remains unknown (80–82).

Further investigation is required to draw concrete conclusions on the efficacy of tDCS. While the applications of this therapy are incredibly wide, limitations still exist with patients who experience chronic pain without impaired CPM, not responding to tDCS (82). The correlation between altered CPM and increased tDCS-related changes further emphasizes the personal experience of treatment responses, with authors highlighting the need for an individualized therapeutic approach to resolving impairments in neuromodulation (53, 77, 82). Given the variability in descending pain modulation across individuals, clinical protocols should integrate neuroimaging or neurophysiological assessments to develop more effective interventions (83). While some current evidence supports its use, inconsistencies in the methods of these studies including differences in stimulation parameters, duration, and outcome measurements demonstrate the need for standardized protocols (84).

Spinal cord stimulation (SCS) is an established therapy for chronic neuropathic pain which was highlighted by many of the reviewed studies. The therapy applies mild electrical impulses to the spinal cord via an implanted electrode which works based on the gate control theory of pain (45, 85, 86). Electrical impulses stimulate A*β* fibers in the dorsal columns of the spinal cord, activating inhibitory interneurons that suppress nociceptive C-fiber input, dampening pain (57, 85). Tonic stimulation, a form of SCS which delivers continuous low-frequency pulses, primarily modulates lateral pain pathways. These pathways encode sensory aspects of pain including location or temperature and show reduced activity in regions such as the thalamus and primary sensorimotor cortex during SCS, according to an EEG study by De Riddler et al. (86). However, delivering stimulations in short bursts, known as BurstDr stimulations, may prove more effective than the traditional methods. Work by Yearwood et al. found that burst stimulations specifically reduce activity in the dACC, a region involved in attention and salience to pain. They also report burst stimulations reduce activity in the posterior cingulate cortex (PCC), which is linked to pain rumination and regulation of the pain setpoint (57). This finding is replicated by De Riddler et al., who found a similar effect in the dACC and PCC, as well as activation in the pregenual anterior cingulate cortex which is believed to exert top-down inhibitory control over the dACC (86). Overall, SCS has been shown to regulate the sensory and emotional components of pain through widespread effects on the brain's pain network and shows promise as a potential therapeutic for chronic pain conditions (45, 57, 86).

Additionally, advances in functional neuroimaging have provided a more detailed understanding of pain network connectivity in populations, emphasizing the potential for biomarkers to predict treatment response (87). The identification of cortical and subcortical pain network changes has resulted in growing interest in individualized treatment approaches that integrate both objective neuroimaging markers and subjective pain assessments (87). These developments are promising for improving decision-making in clinical practice and optimizing the effectiveness of therapies.

These findings support the growing recognition that pain modulation is a complex, multi-system process influenced by neuroplasticity, disease pathology, and psychological factors. Thus, clinicians should adopt a multimodal approach that combines pharmacological, neuromodulatory, and behavioral strategies to maximize therapeutic outcomes. Future research should also explore the long-term implications of neuromodulation and how adaptive changes in pain networks may sustain or diminish therapeutic benefits over time.

## 4 Discussion

The works reviewed have significantly advanced our understanding of descending pain modulation and the factors that influence its control. The literature consistently highlights the pivotal role of the PAG and other brainstem structures like the RVM and its substituents, detailing their function as the central hub for pain modulation through descending inhibitory and faciliatory pathways. In contrast, higher order cortical regions including the prefrontal cortex, anterior cingulate cortex, insular cortex, and amygdala exhibit more variable and complex contributions to pain modulation. Functional and structural studies agree that cognitive strategies like distraction tasks, emotional imagery, or music affect descending pain modulation via altering connectivity between these cortical regions. Maladaptive cognitive processes like pain catastrophizing and anxiety appear to impair descending pain control, disrupting functional connectivity between these higher brain regions and the brainstem and increasing sensitivity to pain. Their involvement appears more context dependent, influenced by cognitive, emotional, and environmental factors, explaining why they do not exhibit a singular function across studies. The applications of this knowledge are also highlighted by the pool of studies which evaluate existing therapies and identify targets for novel ones as well. TDCS was the most covered therapy out of all and shows significant progress as a potential therapy for pain, with the reviewed studies expanding our understanding of how this treatment works and the neurological pathways it affects.

While the reviewed works provide interesting new insights on the function of many brain regions and pathways, researchers often relied on animal models to infer mechanisms of descending pain in humans. While these models provide a strong foundation for our understanding of neural pathways, they may not be able to fully capture the cognitive and emotional influences on pain that occur in human participants. Future studies aimed at validating these findings in human populations will help clarify the nuances of descending pain which cannot be evaluated in animals. Furthermore, while tDCS shows promise as an effective therapeutic for chronic pain diseases, studies often made broad implications of the uses of this treatment. Further research examining tDCS in larger, more diverse study groups over a longer period of time will assess the durability of the treatment as well as its safety and long-term effects. Finally, CPM is often assumed to demonstrate descending pain regulation while the mechanism controlling this process is still being investigated. Many studies have shown that when a test stimulus and conditioning stimulus are simultaneously applied, a significant number of participants do not experience the CPM effect (88–90). While the concept of pain inhibiting pain has been shown in shown in diffuse noxious inhibitory control studies in animals, these effects may not be generalizable to humans who have a wide range of factors influencing the experience of pain (91).

One notable methodological limitation is that the review is restricted to PubMed, raising the possibility that studies that are not included in this database were not captured in the review. However, given PubMed's extensive coverage of neuroscience research, it is unlikely that inclusion of additional databases would have significantly altered the major patterns and conclusions included in this review.

Altogether, these findings highlight both the progress and limitations in our understanding of descending pain modulation. While key brain regions and novel therapies have been identified, further research, particularly in human populations, is required to fully understand these systems. Addressing these gaps will be critical for developing more effective and individualized pain management strategies.
